# Supplementary material for: Identification of Freezing-Responsive microRNAs and Their Targets in Chinese Jujube by Small RNA and Degradome Sequencing
Source: Int J Mol Sci. 2026 Sep 4;27(17):7912. doi: 10.3390/ijms27177912 (PMC13566890; doi:10.3390/ijms27177912)
Supplement: Supplementary file 1 [file ijms-27-07912-s001.zip › Supplementary Material S2/novel_miR_225.pdf]

Provisional ID : novel_miR_225
Score total : 0.5
Score for star read(s) : -1.3
Score for read counts : 0
Score for mfe : 0.2
Score for randfold : 1.6
Score for cons. seed :
Total read count : 64
Mature read count : 64
Loop read count : 0
Star read count : 0

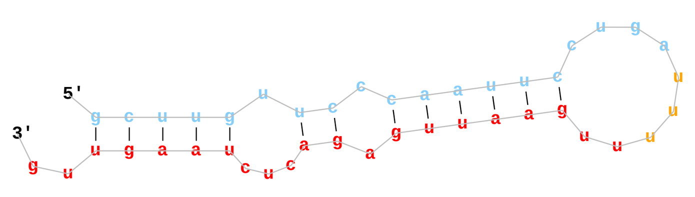

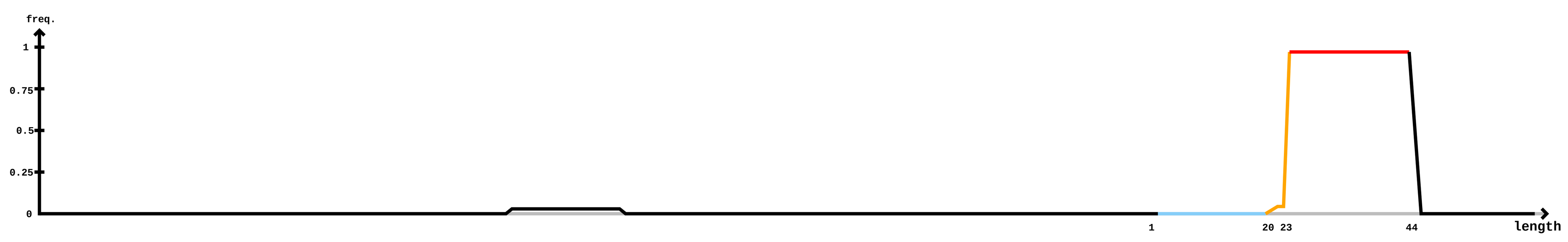

Star Mature

```
5'- aucuuaguuuaacuaaaaaaaauucagagaauagucugaaauauagaugaucuaagugauauuuuaguucauuuuuauugaucuguuagaaagaaacugaaguaauauauaaacucagaugcaagggagagauuucaaucucuccugcuguuugauguuuguuucauuuugauuaaauguaguagugcuuguucccaauuccugauuuuugaauugagacucuaaguugugcacguggaugguaagggaca -3'   exp
    .(((((.(....(((......((((((......)))))).((((..(((((.(((((((((((((((..((((((.........))))))..)))))))))))....((((((((((((((..((((((((....)))))))))))).))))))).))))))).))))))).)))))..))))(((..(((((.((.((((((.........)))))).))...)))))..)))...)))..).)))))....   reads   mm   sample
    ..............................................................................ugaucuguuagaaagaaac..........................................................................................................................................................   1   0   JCK-1
    ..............................................................................ugaucuguuagaaagaaac..........................................................................................................................................................   1   0   JCK-3
    .............................................................................................................................................................................................................uuuuugaauugagacucuaaguug......................   3   0   LT-3
    ................................................................................................................................................................................................................uugaauugagacucuaaguug......................   23   0   LCK-2
    ................................................................................................................................................................................................................uugaauugagacucuaaguug......................   40   0   LCK-1
    ................................................................................................................................................................................................................Cugaauugagacucuaaguug......................   1   1   LCK-1
```
